# Hard on Coroners

**Published:** 1874-01

**Authors:** 


Hard on Coroners.—In a conversation recently, during which something was remarked concerning the death of the old year, we heard a gentleman express the opinion that probably the Buffalo Coroners had not heard of it yet, or they would have held an inquest before this time. In view of the zeal and promptness with which these gentlemen generally perform their *duty* it seems a little severe that any insinuations should be thrown out against them, if they do now and then neglect to hold an inquest over the remains of the departed.

